# Focused stimulation of dorsal versus ventral subthalamic nucleus enhances action–outcome learning in patients with Parkinson’s disease

**DOI:** 10.1093/braincomms/fcae111

**Published:** 2024-04-02

**Authors:** Andrew Willett, Scott A Wylie, Jessica L Bowersock, Benoit M Dawant, William Rodriguez, Beatrice Ugiliweneza, Joseph S Neimat, Nelleke C van Wouwe

**Affiliations:** Department of Neurological Surgery, University of Louisville, Louisville, KY 40202, USA; Department of Neurological Surgery, University of Louisville, Louisville, KY 40202, USA; Department of Neurological Surgery, University of Louisville, Louisville, KY 40202, USA; Department of Electrical and Computer Engineering, Vanderbilt University, Nashville, TN 37235, USA; Department of Electrical and Computer Engineering, Vanderbilt University, Nashville, TN 37235, USA; Department of Neurological Surgery, University of Louisville, Louisville, KY 40202, USA; Department of Neurological Surgery, University of Louisville, Louisville, KY 40202, USA; Department of Neurological Surgery, University of Louisville, Louisville, KY 40202, USA

**Keywords:** subthalamic nucleus, deep brain stimulation, action–outcome learning

## Abstract

Deep brain stimulation of the subthalamic nucleus is an effective treatment for the clinical motor symptoms of Parkinson’s disease, but may alter the ability to learn contingencies between stimuli, actions and outcomes. We investigated how stimulation of the functional subregions in the subthalamic nucleus (motor and cognitive regions) modulates stimulus–action–outcome learning in Parkinson’s disease patients. Twelve Parkinson’s disease patients with deep brain stimulation of the subthalamic nucleus completed a probabilistic stimulus–action–outcome task while undergoing ventral and dorsal subthalamic nucleus stimulation (within subjects, order counterbalanced). The task orthogonalized action choice and outcome valence, which created four action–outcome learning conditions: action–reward, inhibit–reward, action–punishment avoidance and inhibit–punishment avoidance. We compared the effects of deep brain stimulation on learning rates across these conditions as well as on computed Pavlovian learning biases. Dorsal stimulation was associated with higher overall learning proficiency relative to ventral subthalamic nucleus stimulation. Compared to ventral stimulation, stimulating the dorsal subthalamic nucleus led to a particular advantage in learning to inhibit action to produce desired outcomes (gain reward or avoid punishment) as well as better learning proficiency across all conditions providing reward opportunities. The Pavlovian reward bias was reduced with dorsal relative to ventral subthalamic nucleus stimulation, which was reflected by improved inhibit–reward learning. Our results show that focused stimulation in the dorsal compared to the ventral subthalamic nucleus is relatively more favourable for learning action–outcome contingencies and reduces the Pavlovian bias that could lead to reward-driven behaviour. Considering the effects of deep brain stimulation of the subthalamic nucleus on learning and behaviour could be important when optimizing stimulation parameters to avoid side effects like impulsive reward-driven behaviour.

## Teaser

Willett *et al*. investigated the effect of deep brain stimulation in subregions of the subthalamic nucleus on action–outcome learning in Parkinson’s disease. We demonstrated that focused stimulation of the ventral relative to the dorsal subthalamic nucleus led to increased reward-driven learning and a reduction in the ability to learn action–outcome associations.

## Graphical Abstract

**Graphical Abstract:**
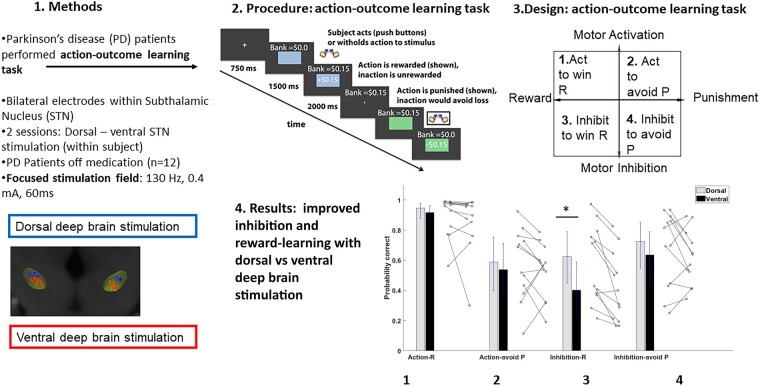

## Introduction

Adapting behaviour in novel environments often requires learning new associations between stimulus events, and how to react to these events, to produce desired outcomes.^1,2^ However, the discovery of these often subtle or unpredictable patterns in everyday life plays an important role in decision optimization, generally to maximizing positive outcomes and minimizing negative ones.

A unique feature of stimulus–action–outcome learning is the demonstration of innate biases between actions and outcomes across species. Such biases, termed Pavlovian biases, include ‘act to gain a positive outcome’ (action–gain reward bias) and ‘inhibit action to avoid a negative outcome’ (inhibit–punishment avoidance bias).^1,3,4^ These action–reward and inhibit–punishment avoidance biases are argued to facilitate decision-making in novel situations by quickly determining which actions should be selected to produce desired outcomes and which actions should be inhibited to avoid undesired outcomes.^5,6^ However, these Pavlovian biases come with trade-offs that can disrupt learning flexibility, particularly the learning of alternative contingencies that violate or conflict with these biases. For example, a strong action–reward bias makes it more difficult to learn a link between inhibiting an action to gain a reward. Similarly, a strong inhibition–punishment avoidance bias makes it more difficult to learn a link between selecting an action to avoid punishment.^1,5,6^

The formation and expression of stimulus–action–outcome associations may be in part organized within frontal–basal ganglia circuitry.^7,8^ A range of neurological and neuropsychiatric disorders associated with altered frontal–basal ganglia circuitry (e.g. Parkinson’s disease, obsessive–compulsive disorder and schizophrenia)^9-11^ experience difficulties learning contingencies between actions and outcomes or, specifically, demonstrate changes in the strength of Pavlovian biases during stimulus–action–outcome learning.^12,13^ In individuals with Parkinson’s disease, a reduction of dopaminergic activity in the basal ganglia shifts learning biases by weakening the action–reward Pavlovian bias while enhancing action–punishment avoidance learning.^14^ Thus, Parkinson’s disease patients (off medication) become more sensitive to actions that help avoid negative outcomes as opposed to actions that generate rewards. Treatments for the clinical motor symptoms of Parkinson’s disease, like dopaminergic medication or deep brain stimulation of the subthalamic nucleus (DBS-STN), appear to restore or even amplify the stronger action–reward Pavlovian bias,^15-17^ which may explain some of the treatment-related increase in reward-driven impulsive behaviour.^18,19^

Given indications that basal ganglia circuitries are implicated in stimulus–action–outcome processing, the current study aimed to advance our understanding of how DBS, directed at specific subregions and the associated circuitries within STN, impacts Pavlovian learning biases and shifts action–outcome sensitivities during learning. A clearer picture of STN subregion and circuitry effects could also contribute novel insights about behavioural side effects of DBS (e.g. impulsivity, apathy) as well as inform electrode placement decisions to minimize certain cognitive side effects.

DBS therapy for Parkinson’s disease offers a unique opportunity to probe links between basal ganglia function and stimulus–action–outcome learning. With DBS, specific structures, like the STN or the globus pallidus interna of the basal ganglia, can be stimulated directly with high-frequency electrical pulses. The STN may play a particularly unique role in action–outcome decisions and learning because of its putative contribution to the inhibition of action.^20^ The STN is embedded in a frontal–striatal circuitry inclusive of presupplementary motor area (preSMA), inferior frontal cortex (IFC), insula and anterior cingulate cortex (ACC) that coordinate action selection and suppression mechanisms.^20-25^ Stimulating the STN reduces action thresholds in a global manner, thus restoring Parkinson’s disease patients’ ability to move more freely but also increasing their risk of reacting prematurely or impulsively.^26^ It has been proposed that when a conflict between action choices arises, the cortex activates the STN through the hyper-direct pathway.^27^ The excitation of STN suppresses basal ganglia output to the thalamus, which stops or pauses action-generating signals from triggering motor responses in the cortex.^20,28^ In Parkinson’s disease, the overactive STN induces a strong braking of action that is alleviated by DBS-STN. Support for this idea comes from studies investigating the effects of DBS-STN and of STN-lesioned animals that have reported a DBS-induced increase in fast, premature response errors.^29-36^ This suggests that lowering the threshold to act with clinical DBS during reward–learning or reward-based decision-making may thereby amplify the Pavlovian action–reward bias.

While clinical DBS-STN generally targets dorsal motor areas of the STN (innervated by primary motor cortex and supplementary motor cortex), a confounding factor in using clinical DBS parameters is that they produce large stimulation fields, thereby impacting multiple STN subregions and subcircuitries.^22,37^ The potential impact of stimulation in STN subregions on learning that involves both action selection and inhibition, as well as processing reward and punishment outcomes, is largely unexplored. There may be dissociable effects on stimulus–action–outcome learning when focal stimulation is directed to either dorsal motor areas of the STN or the relatively ventral cognitive and limbic STN subregions.

The ventral STN subterritories are innervated by preSMA, IFC, dorsolateral prefrontal cortex, ACC and orbital frontal cortex^22^ and connected to a range of cognitive and emotional processes and effects.^38-41^ Stimulation of ventral STN circuitries has been shown to impact the processing of emotional valence and may also modulate the appraisal of positive or negative outcomes.^41,42^ Thus, specific effects of DBS-STN on action–outcome processing may depend on the subregion stimulated. In the current study, we refer to dorsal and ventral subregions of the STN, but note that this is synonymous to the superior versus inferior direction along the STN.

A thorough investigation into how DBS targeting distinct STN subterritories influences bias in learning stimulus–action–outcome associations will enhance our understanding of the role of STN in outcome-based learning in general and also contribute to a deeper understanding of the underlying processes that might relate to some of the unintended side effects of STN stimulation. In this study, we investigated how subregional DBS-STN impacts stimulus–action–outcome learning by measuring learning effects when stimulation was being delivered to electrodes positioned in relatively dorsal STN versus relatively ventral STN. Based on the dissociable functional inputs to STN subregions, we hypothesized that stimulation of the relatively dorsal, motor subregions of the STN would exert stronger effects on the action component of action–outcome learning (action selection versus action inhibition) while stimulation of the relatively ventral STN subregion would exert specific effects on the outcome or valence (reward versus punishment avoidance) component of action–outcome learning.

## Materials and methods

### Participants

Parkinson’s disease participants (*n* = 12), who had previously undergone bilateral DBS-STN implantation, were recruited from the Movement Disorders Clinic at Vanderbilt University Medical Center. All participants were withdrawn from dopaminergic medications during study participation following a 24-h withdrawal from levodopa and 48-h withdrawal from dopamine agonist.

Inclusion criteria included idiopathic Parkinson’s disease with bilateral STN implants, with lead contacts localized in dorsal and ventral subregions of STN (see ‘DBS contact registration and selection’ for details).

Exclusion criteria were similar to previously published studies^43,44^ and are detailed in the Supplementary material. Patients were recruited after DBS surgery based on electrode positioning methods described below. Prior to study enrolment, participants experienced a minimum of 6 months’ improvement in clinical motor symptoms as classified by medical record review and neurological ratings of motor symptoms [Movements Disorders Society Unified Parkinson’s Disease Rating Scale Motor (MDS UPDRS-III)]. On average, participants were recruited 26 months post-surgery (SE = 5.3 months). Individual demographic and clinical profiles are outlined in Table 1. Clinical stimulation parameters are shown in the Supplementary Tables 1 and 2.

**Table 1:** Demographic and clinical data (means and standard deviation)

Demographics	
Sample size (*N*)	12
Age (years)	59 (2.40)
Sex (M:F)	8:4
Education (years)	15.29 (1.02)
MMSE	28.42 (0.43)
CESD	14.58 (2.57)
BIS-II	61.42 (3.27)
LEDD^a^	463.33 (123.02)
Disease duration (years)	14.13 (2.19)
UPDRS-III dorsal^b^	27.8 (2.19)
UPDRS-III ventral^b^	28.0 (2.68)
UPDRS-III preop OFF DA (*n* = 10)	45.2 (2.65)
UPDRS-III preop ON DA (*n* = 9)	22.0 (2.44)

MMSE, Mini-Mental State Examination; CESD, Center for Epidemiologic Studies Depression Scale; BIS-II, Behavioral Impulsivity II; LEDD, levodopa daily dose; UPDRS, Movement Disorders Society Unified Parkinson’s Disease Rating Scale Motor. Dorsal and ventral UPDRS were collected during the research visit with focused stimulation settings. UPDRS preop was captured before surgery (ON and OFF dopaminergic medication). ^a^No significant difference between dorsal and ventral DBS (*P* > 0.94). ^b^Focused dorsal and ventral stimulation significantly improved UDPRS compared to preoperative UDPRS ON and OFF DA (*t*s > 2.4, *p*s < 0.04).

A detailed informed consent process was conducted before enrolment. Research activities complied with the standards of ethical conduct in human subject research as outlined by the Vanderbilt University Medical Center.

### DBS contact registration and selection

DBS contact registration and selection were performed using methods similar to our previous studies.^43,44^ Per standard clinical care, participants underwent preoperative MRI with T_1_-weighted and T_2_-weighted images, which were acquired via 3T Philips (Philips Achieva) using phased array SENSE 8-channel reception and body coil transmission. We captured T_1_-weighted images [typical time repetition (TR)/time echo (TE) = 7.9/3.6 ms] using 1.0 mm^3^ isotropic spatial resolution and captured T_2_-weighted images (typical TR/TE = 3000/80 ms) using a 47 × 47 mm^2^ in-plane resolution and 2 mm slice thickness. Postoperative imaging (1 month after surgery to allow for resolution of pneumocephalus) included a brain CT collected at kVp = 120 V, with 350 mAs exposure, capturing 512 × 412 pixels. In-plane resolution was set at 0.5 mm while slice thickness was set at 0.75 mm. Note that patients were not rescanned prior to study enrolment, but we have previously shown that DBS leads secured with our surgical technique do not significantly migrate.^45^

We localized DBS contacts both manually and automatically using the CRAnialVault Explorer (CRAVE^46^) software, as described previously.^43,44^ Briefly, we used the software to first localize DBS implants and contacts in the CT images (delayed ∼4 weeks to allow resolution of pneumocephalus) and then visually verified the localization accuracy for each individual contact. To determine the anatomic location of each contact, the preoperative MR images were first registered to the postoperative CT images with the use of fully automatic intensity-based rigid registration techniques integrated into CRAVE. Next, the CRAVE T_1_-weighted MR anatomic atlas in which deep brain structures including the STN have been localized using high-field (7 Tesla) images^47^ was registered to the preoperative T_1_-weighted image. This was achieved with a fully automatic intensity-based nonrigid technique also integrated into CRAVE.^48^

Contours of anatomic structures were projected from the atlas to the preoperative T_1_-weighted images, and registration accuracy was assessed visually. If either contact localization or atlas registration was deemed inaccurate, the subject was excluded from the study. Ultimately, individual contacts were projected onto the atlas using the inverse of the atlas-to-subject transformation. The accuracy of this last step was again assessed visually by confirming that the contacts were in the same STN subregion in the atlas and the subject’s T_1_-weighted image.

**Figure 1:**
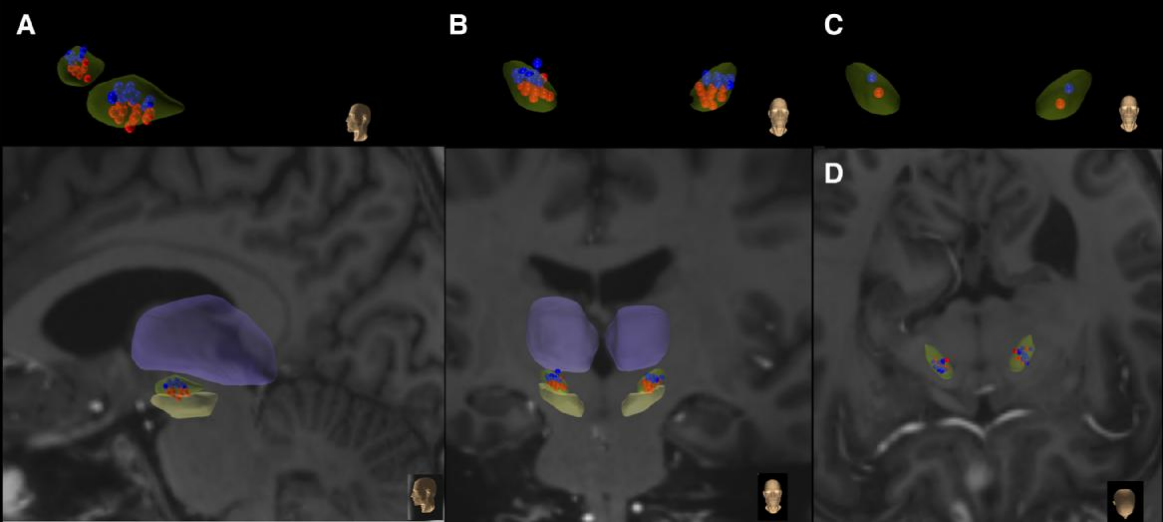
**Individual electrode positions in subthalamic nucleus (STN) used for dorsal (blue) and ventral (red) stimulation in (A) sagittal (enlarged and overview), (B) coronal planes (enlarged and overview) and (C) average electrode position in coronal plane and (D) individual electrodes in axial plane.** Substantia nigra (structure below STN) and thalamus (structure above STN) are displayed for reference. Electrode volumes represent the estimated VTA radius, i.e. based on Butson and McIntyre,^49^ the projected volume of tissue activation with 0.4 mA, 130 Hz and 60 ms (with an average clinical impedance of 1 kΩ) would result in a VTA radius of ∼1.3 mm.

Figure 1A–C show the individual electrodes used for dorsal and ventral stimulation projected onto the atlas STN. Figure 1D shows the groups’ average position. As was done in our previous study,^43^ for each individual, we defined the ventral and dorsal STN using an oblique plane (perpendicular to the lead trajectory) and further categorized the dorsolateral and ventromedial subregions in line with Haynes and Haber.^22^ Participants were deemed appropriate candidates for recruitment if bilateral leads had a minimum of one contact in both the dorsal and ventral STNs (as defined by their individual scan mapped on the atlas in CRAVE). Table 2 shows the electrodes that were used for focused stimulation at dorsal and ventral contacts.

**Table 2:** Electrodes used for the experimental focused stimulation at relative dorsal and ventral contacts

	Dorsal contact		Ventral contact	
Subject ID	Left	Right	Left	Right
**1**	3	3	2	2
**2**	3	3	2	2
**3**	2	2	1	1
**4**	1	1	0	0
**5**	2	3	0	2
**6**	2	3	1	2
**7**	1	1	0	0
**8**	2	2	1	1
**9**	2	2	1	1
**10**	3	3	2	2
**11**	2	2	0	0
**12**	3	2	1	0

Experimental bilateral stimulation settings were set at 0.4 mA, 130 Hz frequency and 60 ms pulse width. Note 1: for Medtronic 3389 leads, 0 indicates the most ventral lead and 3 is the most dorsal lead of the four-contact array. The 3389 leads have an electrode contact size of 1.5 mm with 0.5 mm spacing between contacts. Note 2: subjects 5 (left side), 11 and 12 did not receive ventral/dorsal stimulation at adjacent contacts, and the ventral contact subject 11 (right) and subject 12 (left) were partially inside the STN. All other participants received stimulation at a ventral contact immediately below a dorsal contact.

### Design and procedures

Participants completed two sessions of a stimulus–action–outcome learning task,^1,6,15,16,50^ a session with dorsal STN stimulation and a session with ventral STN stimulation. The study was limited to two stimulation settings (no ‘OFF’ condition included) to limit the overall experiment time and discomfort for the patients. The order of sessions was counterbalanced across participants, and participants were blinded to the order of stimulation. Before the first testing session and between DBS setting changes, we included a 30-min waiting period. Prior studies suggest this time period is sufficient to produce dissociable effects on cognitive and action control processes^16,44^ and accounts for most of the changes in the motor symptoms.^51,52^

The participants’ clinical DBS settings were modified to specifically target STN subregions by adjusting the stimulation parameters. Unlike the clinical DBS settings, which often lead to overlapping stimulation fields across adjacent contacts, we maintained a consistent current of 0.4 mA, a stimulation frequency of 130 Hz and a pulse width of 60 μs.^43,44^ These settings aimed to achieve a uniform current density throughout the targeted STN subregions and across all participants while limiting the estimated field of tissue activation. According to Butson and McIntyre,^49^ applying a stimulation amplitude of ∼0.4 mA (assuming an average clinical impedance of 1 kΩ) would result in a volume of tissue activated (VTA) with a radius of about 1.3 mm. This approach ensured minimal volumetric overlap between adjacent electrodes to ensure distinguishable effects.

For the learning task, participants were informed that their goal was to learn whether to act or inhibit action to each of four different colour patches to maximize monetary earnings, either by gaining a reward or avoiding a loss (punishment). This task has been widely used to measure Pavlovian biases in learning, to contrast action versus inhibition learning and to investigate the role of reward versus punishment avoidance in learning.^1,6,15,16,50^

The task started with the presentation of a centred fixation point for 750 ms. After the fixation point extinguished, one of the four colour patches flashed on the screen for 200 ms. Participants then had 2 s to either act (i.e. make a two-handed button press; the bilateral response avoids potential handedness effects) or inhibit action (i.e. withhold or refrain from the two-handed button press). After the 2-s response decision window, feedback was displayed on the screen for 2 s and indicated if the participant’s action choice (act or inhibit action) resulted in one of three outcomes: monetary reward (+ 25 cents), monetary loss (− 25 cents) or no monetary outcome (0 cents). The feedback information then disappeared, triggering the next trial with presentation of one of the colour patches. For each session, a set of four unique colour patches appeared in random order with equal probability (10 times for each colour) across 4 blocks of 40 learning trials. The order of these sets of colour patches was counterbalanced across subjects.

Within a session, each colour was presented 40 times across the 4 blocks. Throughout each block, participants could see their total earnings displayed in the upper centre of the screen. One individual block lasted for ∼3 min, and 1-min breaks were allotted between blocks. Before the experiment started, they were given 15 practice trials.

As the participants were blinded to which colours were associated with the respective outcomes, they were tasked to learn to maximize their outcomes by optimizing reward and avoiding losses. Additionally, two colours from each set produced the ideal outcome by acting (either gaining a reward or avoiding punishment), while two others produced the ideal outcome via inaction. This 2 × 2 factor design setup allows for evaluation of action and valence.

However, patterns were not 100% deterministic throughout the learning blocks to keep the task engaging and challenging, thereby maintaining the participants’ attention. Participants were instructed that each stimulus–action–outcome association was probabilistic and would yield the desired outcome most of the time but not all of the time. The stimulus–action–outcome conditions are outlined below, and the optimal response that would lead to higher accuracy and more reward/fewer losses for each stimulus is underlined.

1. Stimulus A: action to gain reward. Participants had a 90% chance of receiving a reward with an action to this stimulus and only a 10% chance that action was not rewarded. Inhibiting or withholding action was not rewarded 100% of the time.
2. Stimulus B: inhibit action to gain reward. Participants had a 90% chance of receiving a reward when they inhibited or withheld action and only a 10% chance that inhibiting or withholding action was not rewarded. Acting to this stimulus was not rewarded 100% of the time.
3. Stimulus C: action to avoid punishment. Participants had a 90% chance of avoiding a negative outcome (no loss) when they acted to this stimulus and only a 10% chance of receiving a negative outcome (loss of $) when they acted. Inhibiting or withholding action to this stimulus resulted in a negative outcome 100% of the time.
4. Stimulus D: inhibit action to avoid punishment. Participants had a 90% chance of avoiding a negative outcome (no loss) when they inhibited or withheld action to this stimulus but only a 10% chance of receiving a negative outcome (loss of $) when inhibiting or withholding action. Action to this stimulus led to a negative outcome 100% of the time.

### Behavioural outcome measures

Participants performed 160 learning trials in total, 40 trials for each of the 4 unique colour patches. Our primary behavioural outcome was accuracy across trials and conditions. Higher accuracy (through action or inaction) on the conditions reflects better learning. We also collected reaction times (RTs) for the trials that required an action, although speed was not emphasized in the task instructions.

Additionally, we calculated a Pavlovian reward or punishment bias similar to previous studies.^15^ Specifically, the error rate on trials with ‘conflicting’ action–outcome associations (action–punishment avoidance, inhibition–reward) was divided by the accuracy on the Pavlovian bias conditions separately for reward and punishment conditions. The reward bias was calculated by dividing the errors on ‘inhibition–reward’ trials by the accuracy on the ‘action–reward’ trials. The avoid punishment bias was calculated by dividing errors on ‘action–avoid punishment’ trials by the accuracy of the ‘inhibition–avoid punishment’ trials. The numerator represents the impact of the Pavlovian biases on learning the unnatural or conflicting stimulus–action–outcome conditions, which is scaled by the denominator (reflecting the strength of learning the more natural or Pavlovian associations). A smaller value on the Pavlovian bias (closer to 0) indicates fewer errors on unnatural associations during the task, whereas values closer to 1 indicate a higher Pavlovian bias; i.e. more errors were made on unnatural associations due to the bias of responding in line with the expected natural action–valence combinations. A large number of errors on the inhibition–reward trials relative to the accuracy on ‘action–reward’ trials would thus result in a high reward bias (close to 1). Similarly, a high punishment bias would be the result of a high error rate on the action–avoid punishment trials relative to the accuracy on inhibition–avoid punishment trials.

### Statistical techniques

We used a generalized mixed linear model (GLM) with a binary distribution and a logit link to test whether the probability of an accurate response depended on the STN subregion, action or outcome condition. Independent variables were DBS subregion (dorsal STN, ventral STN), action choice (action, inhibit action) and outcome (reward, avoid punishment) and the interactions between these variables. We also included the independent variable trial number (1–40) and the interaction of trial number with DBS, action and valence to capture effects across trials. A random intercept per individual was included to account for individual variability for each subject. In addition, a hierarchal design was specified by adding random effects of trial number nested within the interaction of action and outcome (action and outcome nested within DBS) for each individual subject. Logistic regression model assumptions^53^ (absence of multicollinearity, linearity of logit with the continuous variable trial number, lack of strongly influential outliers and the independence of errors) were checked. Biserial correlations were used to evaluate the correlation between trial number and the other independent variables (all binary). The linearity of logit accuracy and the continuous variable trial number was evaluated by a visual evaluation of the scatter plot trial number versus residuals. Different dependencies due to the longitudinal nature of the experiment were adjusted for through the hierarchical/nested/mixed model design. The significance level was set to 5%.

Additionally, we analysed whether there was an effect of STN subregion stimulation on RTs for the action trials. We compared the RTs for the stimulus–action–outcome conditions (action–reward, action–avoid punishment) between STN subregions with a repeated measures (RM) ANOVA, within subjects’ factors outcome and DBS. We used a more parsimonious ANOVA model for the RTs rather than a GLM because (i) the RTs were only available for the learning trials that required an action (half of the trials), and (ii) the learning task instructions did not emphasize speed; thus, we did not have specific predictions about RTs across trials that would require a multifactorial model.

Pavlovian biases during dorsal and ventral DBS-STN were analysed with repeated sample Wilcoxon rank order tests (due to their skewed distribution), separately for reward and punishment–avoidance bias.

Statistical analyses were performed in SAS 9.4 (SAS Inc., Cary, NC), R 4.1.12^54^ and SPSS 29 (IBM Statistics).

## Results

### DBS-STN subregion effects on action–outcome learning performance (accuracy)

**Figure 2:**
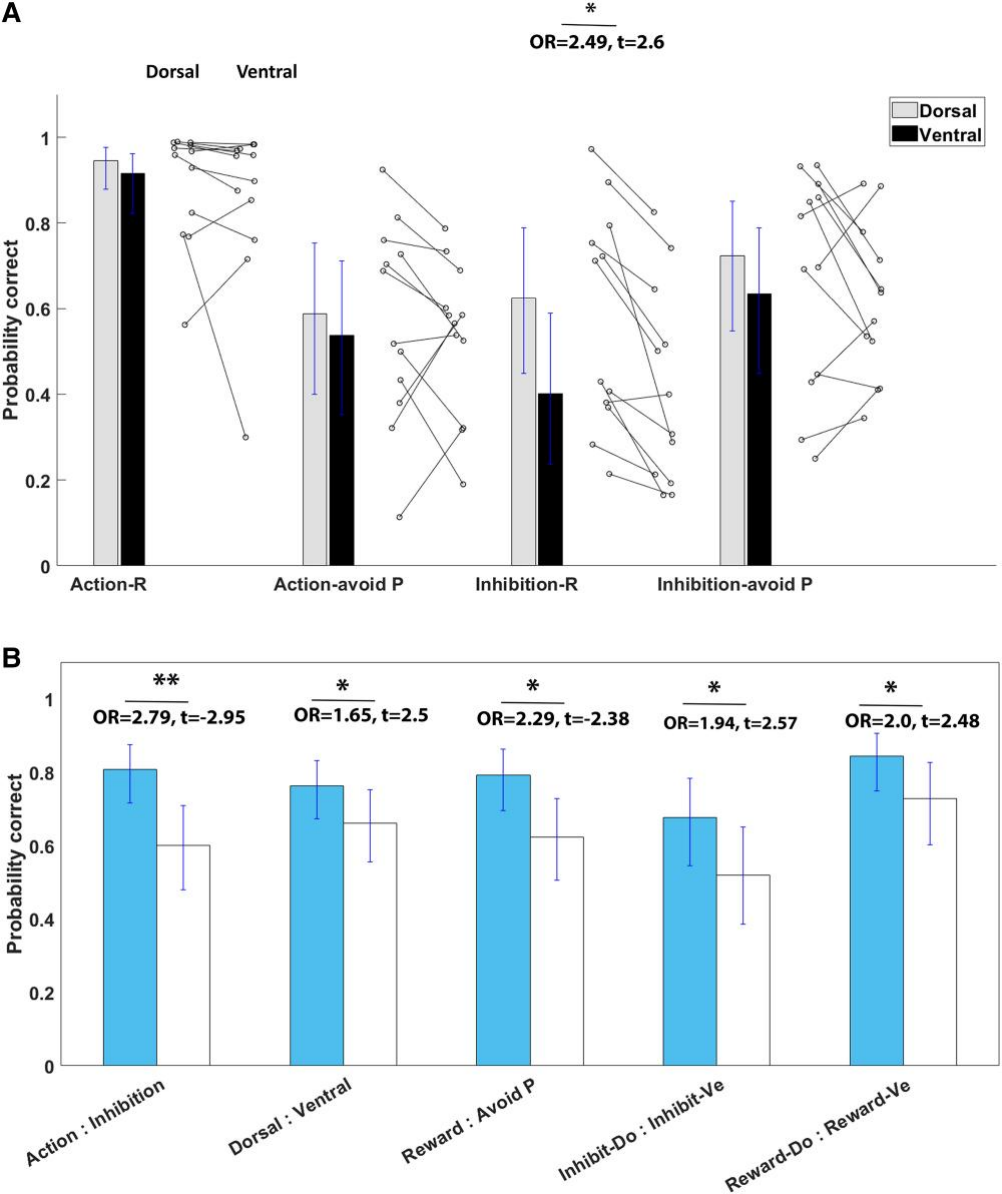
**(A) Estimated probability of a correct response by learning condition and DBS.** Lines within the bars reflect 95% confidence intervals. Connected dots next to the bars show individual variability in performance with dorsal and ventral stimulation for each condition. (**B**) Estimated probability of a correct response between conditions with significant differences (action, STN subregion, valence and interaction effects between action and STN subregion and between valence and STN subregion) based on GLM (OR > 1.65, *t*s > 2.38, *p*s < 0.05). *P < 0.05; **P < 0.01.

**Figure 3:**
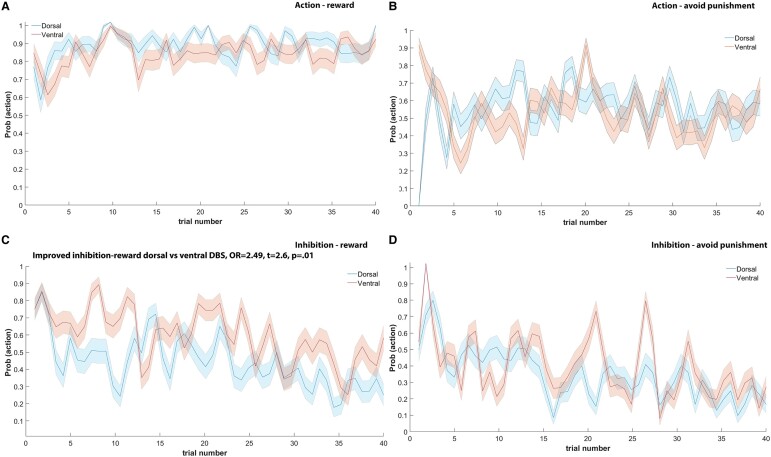
**Trial-by-trial probability that a participant responds with an action for each action–valence condition separated by DBS of the STN site.** (**A**) Action–reward. (**B**) Action–avoid punishment. (**C**) Inhibition–reward. The GLM showed improved inhibition–reward with dorsal versus ventral DBS (OR = 2.49, *t* = 2.6, *P* < 0.05). (**D**) Inhibition–avoid punishment. Note that when learning takes place, the probability to act across trials is expected be high at the end of the 40 trials for the action conditions (**A** and **B**) and expected to be low at the end of the 40 trials for the inhibition learning conditions (**C** and **D**).

The GLM analysis tested the effect of stimulation site and learning conditions (action and outcome) on the probability of an accurate response during the learning task. Figure 2 displays the probability of an accurate response across trials for each stimulus–action–outcome condition, separate for dorsal and ventral STN stimulation and main effects across conditions. Figure 3 shows the detailed trial-by-trial probability that a participant responds with an action for each action–valence condition separated by DBS-STN site. The probability of an accurate response across trials and learning conditions was significantly larger with dorsal DBS-STN (76.36%) compared to ventral STN [66.15%; odds ratio (OR) = 1.65, *t* = 2.50, *P* = 0.03; note that an OR of 1.65 indicates that the likelihood of a correct response with dorsal DBS was 1.65 times greater than with ventral DBS; the significance of these ORs is indicated by the *t*-statistic]. Across DBS conditions, trials associated with action were more likely to receive a correct response (80.77%) compared to trials associated with inhibition (60.05%; OR = 2.79, *t* = −2.95, *P* = 0.004). Moreover, across DBS conditions, responses were more likely to be correct on trials linked to reward (79.19%) compared to trials linked to punishment avoidance (62.39%; OR = 2.29, *t* = −2.38, *P* = 0.02).

In terms of DBS effects, with dorsal STN stimulation, responses were more likely to be correct compared to ventral stimulation on conditions where participants had to learn to inhibit action to produce optimal outcomes (inhibition conditions: dorsal = 67.66%, ventral = 51.92%, OR = 1.94, *t* = 2.57, *P* = 0.01). Additionally, responses were more likely to be correct with dorsal relative to ventral DBS-STN on conditions where participants were optimizing reward outcomes (reward conditions: dorsal = 84.34%, ventral = 72.89%, OR = 2.00, *t* = 2.48, *P* = 0.02). In contrast, the subregional stimulation effect on learning accuracy did not differ in conditions where participants learned to act to optimize outcomes (action conditions: dorsal = 83.29%, ventral = 77.97%, OR = 1.41, *t* = 1.23, *P* = 0.22) or when they learned to optimize decisions to avoid punishment (punishment avoidance conditions: dorsal = 65.95%, ventral = 58.7%, OR = 1.36, *t* = 1.21, *P* = 0.23).

Stimulating STN subregions produced a specific interaction effect. DBS targeting the dorsal STN increased the likelihood of an accurate response when learning to inhibit action for a rewarding outcome (dorsal DBS-STN: 62.5%; ventral DBS-STN: 40.08%; inhibition–reward conditions: OR = 2.49, *t* = 2.6, *P* = 0.01) compared to targeting the ventral portion of the STN, when participants were more likely to experience greater difficulties learning to inhibit action to obtain rewards.

### DBS-STN subregion effects on response times during action learning (RTs)

**Figure 4:**
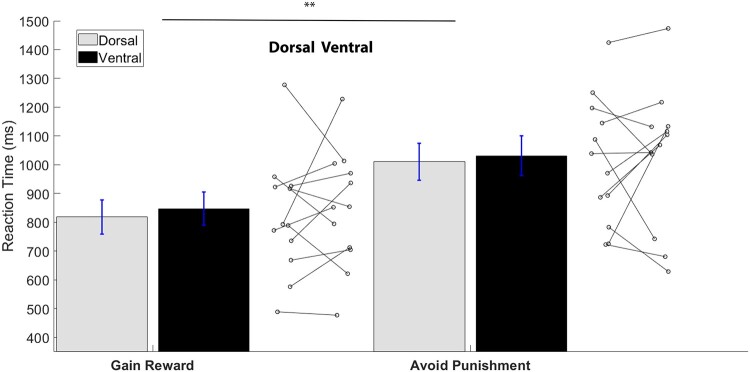
**Average RTs (ms) for action trials separated by stimulation site and outcome.** Overall, performance was faster with the trials that had a rewarding outcome, as shown by the RM-ANOVA [*F*(1,11) = 11.33, *P* < 0.01]. Connected dots next to the bars show individual variability in performance with dorsal and ventral stimulation for each condition. **P < 0.01.

The ANOVA analysis tested whether stimulation site and outcome valence (for the action trials) impacted the RTs on the learning task. Figure 4 displays the mean RTs (ms) for action trials separated by stimulation site and outcome. In conditions requiring learning to act (‘action–reward’ versus ‘action–avoid punishment’), participants’ responses were significantly faster choosing action that produced rewarded outcomes (833 ms) compared to action that led to avoiding punishment or loss [1021 ms; outcome; *F*(1,11) = 11.33, *P* = 0.006, *η*^2^ = 0.51]. There was no effect of DBS subregion [*F*(1,11) = 0.34, *P* = 0.58, *η*^2^ = 0.03] nor an interaction of DBS and outcome (*F*(1,11) = 0.01, *P* = 0.92, *η*^2^ = 0.01) on RTs.

### DBS-STN subregion effects on Pavlovian learning biases

**Figure 5:**
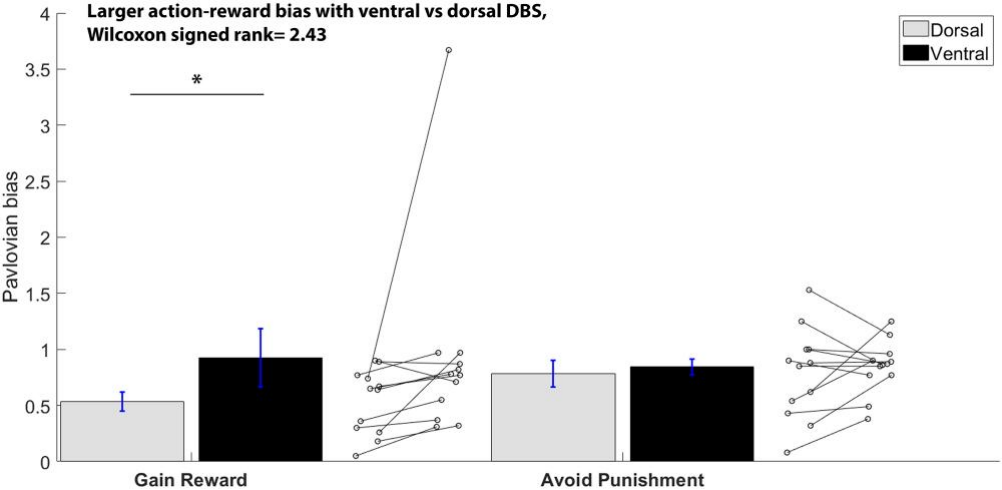
**Average Pavlovian bias separated by stimulation site and outcome.** Ventral stimulation resulted in a larger Pavlovian reward bias relative to dorsal stimulation. Connected dots next to the bars show individual variability in performance with dorsal and ventral stimulation for each bias. Note that removal of the outlier in the ventral reward bias does not change the effect of subregion stimulation, i.e. the reward bias remains significantly larger with ventral than the dorsal DBS (Wilcoxon signed rank = 2.43, *P* = 0.02). *P < 0.05.

The Wilcoxon rank order tests were used to investigate Pavlovian biases by stimulation site and outcome valence. Figure 5 displays the mean Pavlovian bias separated by stimulation site and outcome. Wilcoxon rank order test for paired samples showed a significantly stronger action–reward Pavlovian bias with ventral DBS (0.93) compared to dorsal DBS-STN (0.53), Wilcoxon signed rank = 2.43, *P* = 0.015. That is, with ventral STN stimulation, participants experienced greater difficulty overriding the bias associating action to reward, which made them less effective at learning to inhibit action to produce reward. This aligns with the finding in the mixed model analysis that showed a learning advantage under dorsal STN stimulation for conditions requiring action inhibition to gain reward. Notably, there was no difference in STN subregional stimulation effects on the Pavlovian inhibition–avoid punishment bias, Wilcoxon signed rank = 0.47, *P* = 0.64 (dorsal = 0.78, ventral = 0.84).

## Discussion

We investigated the effects of applying focused stimulation to distinct STN functional subregions on stimulus–action–outcome learning. Parkinson’s disease patients showed expected learning patterns in line with previous findings on similar probabilistic action–outcome learning tasks.^1,6^ That is, participants performed relatively better with stimuli that required action versus inhibition, and they showed more proficient performance with stimuli that led to reward compared to avoiding punishment. Participants also showed Pavlovian biases in their learning patterns. They were most adept at learning to act to obtain a reward and to inhibit action to avoid punishment, a pattern consistent with Pavlovian biases. In contrast, performance was reduced for action–outcome contingencies that conflicted with these Pavlovian biases, such as learning to act to avoid punishment or learning to inhibit action to gain a reward, which replicated prior findings.^1,6^ Specifically, Parkinson’s disease patients showed overall better learning performance when stimulation was bilaterally applied to the dorsal STN compared to the ventral STN. With respect to subregional DBS effects on specific learning patterns and conditions, the most striking differences between DBS-STN subregions were found when learning to inhibit action to optimize outcomes or when learning to gain a reward and particularly when these were combined and required learning to inhibit action to gain rewards. With ventral STN stimulation, learning to inhibit action to obtain reward was markedly less proficient, suggesting a more pronounced Pavlovian action–reward bias that interfered with their ability to associate action inhibition to obtain reward. Stimulating dorsal STN countered this bias and led to more proficient learning to inhibit action to optimize reward.

### Clinical implications of DBS-induced shifts in Pavlovian biases

The relatively stronger action–reward Pavlovian bias with ventral STN simulation compared to dorsal STN stimulation may provide novel clues about alterations in the perception of reward.^55^ In the current study, stimulating ventral STN made it more challenging to learn to inhibit or restrain behaviour to generate rewarding outcomes, suggesting a shift towards a stronger drive towards action in the pursuit of rewards. This is in line with studies showing DBS-STN side effects like euphoria, apathy and impulsivity^56-61^ that could potentially be explained by stimulation of the valence processing network through the more ventral regions of the STN^62,63^ innervated by limbic circuitries.^22,37^

Two prior investigations in Parkinson’s disease studied the effects of clinical DBS settings on a similar action–valence learning paradigm and demonstrated an enhanced Pavlovian action–reward bias when DBS-STN was turned ON compared to OFF.^15,17^ Eisinger *et al*.^15^ also reported a positive relationship between the strength of reward bias and clinical impulsivity measured by the Questionnaire for Impulsive-Compulsive Disorders in Parkinson’s Disease (QUIP^64^). Since clinical DBS settings produce tissue activation fields that easily encompass large sections of STN, it is certainly possible that enhanced action–reward bias was driven by stimulation effects in ventral STN, which would also be consistent with a stronger relationship between induced behaviours captured by the QUIP and stronger action–reward bias. The current study used carefully calibrated stimulation parameters to focus the field of tissue activation to STN subregions (see also van Wouwe *et al.*^43,44^). This more focused approach provides direct confirmation that DBS in ventral STN may be associated with a stronger action–reward bias compared to dorsal stimulation. Dorsal DBS-STN selectively appears to counteract this bias.

It is important to recognize that while the current results implicate a clear dissociation in action–reward bias with ventral STN compared to dorsal DBS-STN, we cannot conclusively state whether ventral STN stimulation led to an increased reward bias or whether dorsal DBS reduced the reward bias because our study did not include a control condition with DBS turned OFF. In a previous comparison between Parkinson’s disease ON and OFF dopaminergic medication on a similar action–valence learning task,^16^ we demonstrated that performance ON medication while learning to ‘inhibit action to obtain reward’ was at 54%; this is relatively higher than what we found in the current study with ventral DBS-STN (40%), but worse compared to dorsal DBS-STN (62.5%). This comparison is at least suggestive that stimulating the ventral, limbic STN subregion could exacerbate the action–reward bias beyond the effect of dopaminergic medication, whereas stimulating the dorsal, motor STN subregion appears to reduce it relative to dopamine medication. Note that the previous study^16^ was performed with different outcome probabilities (80–20 versus the 90–10 used here); thus, an important objective of future research is to investigate the combined effects of medication and DBS on these action–outcome learning biases. Additionally, preoperative individual differences in impulsivity as measured by the QUIP could play an important role in the DBS effect on the reward bias with ventral stimulation and future studies could take this into account.

### DBS-STN subregion effects on inhibitory action and punishment avoidance learning

Beyond the effect of DBS on Pavlovian biases, we had predicted that stimulation of the relatively dorsal STN region might exert stronger effects on the action versus inhibition dimension of stimulus–action–outcome learning. Overall, dorsal DBS-STN was associated with a higher proficiency of learning to inhibit action to optimize outcomes compared to the effects of ventral DBS-STN. This improved inhibition learning was especially effective at countering the strong ‘action–reward’ Pavlovian bias. The relatively dorsal STN substructure is innervated by hyper-direct projections from the supplementary motor area and M1. These cortical–STN circuitries have been associated previously with the inhibition of motor impulses in the Simon task.^65,66^ Similarly, previous DBS effects on an inhibitory action control task (Simon task) demonstrated that focused dorsal stimulation and clinical DBS improved the ability to selectively inhibit undesired action impulses in situations of conflict.^33,43^ Thus, the finding that dorsal STN stimulation appears to play a role in action inhibition learning is consistent with a role in inhibitory motor control more broadly.

It is worth mentioning that stimulating dorsal STN may achieve more proficient inhibition learning through an alternative mechanism. It has been proposed that the STN is involved in setting action decision thresholds, either when regulating action impulses or when learning to associate actions with optimal outcomes.^20,21,26^ Specifically, when conflicts arise between response options, such as between a more optimal action that conflicts with an action consistent with a Pavlovian bias, the dorsal STN may engage a transient inhibition or pause signal to allow more time to select an optimal choice. DBS applied to the dorsal motor network of the STN may be beneficial for disrupting the dysfunctional beta-band oscillations in Parkinson’s disease and thereby improve action related cognitive performance.^67^

Since action inhibition and punishment avoidance learning form the basis of a strong Pavlovian bias, we further speculated that dorsal DBS-STN might impact punishment avoidance learning as well. We did not observe a beneficial effect of dorsal DBS-STN (compared to ventral STN) with respect to the punishment avoidance bias, i.e. on the learning conditions that conflict with the Pavlovian punishment avoidance bias (‘action–avoid punishment’). Participants showed a slightly stronger punishment avoidance bias than a reward bias, and this bias may have been harder to overcome across stimulation conditions. Future EEG imaging studies should aim to establish how DBS-STN (across subregions) impacts the relative amount of conflict induced by the dissociable Pavlovian biases, for example as reflected by a positive or negative prediction error in MPFC or ACC or by the conflict related power in the theta band.^21^

The current study’s observation of improved action inhibition learning with dorsal DBS-STN also seems to contrast with recent findings that ventral stimulation benefits another form of inhibitory control: the proficiency to stop intended actions (as measured by the stop task).^44,68^ Neurophysiological and imaging studies have also associated the ventral STN circuitry to stopping control.^20,69^ However, withholding action in context of the stop task involves exclusively inhibiting a motor response and does not require stopping a valence-induced action bias like the action–outcome learning task. Potentially, in the presence of outcome information, actions might be biased more strongly by their associated valence during ventral relative to dorsal DBS-STN. This could be established by investigating the effect of focused STN subregion stimulation separately for motor and valence conflict, or by parametrically adjusting the relative role of conflicting valence information (for example using variable reward magnitudes).

### Limitations and future studies

We acknowledge that the exact delineation of functional subregions within the STN is still under debate and may display a gradient akin to the structural gradient of cortical connections from motor, associative and limbic areas, spanning the dorsolateral to ventromedial STN.^70,71^ Future studies could benefit from technological advancements in axonal pathway,^72^ normative connectomes^73^ and electrical field modelling^74,75^ to investigate the functional pathways that are impacted by DBS. These developments offer the potential to more precisely locate the DBS effect on functional and structural gradients within the STN circuits that play a role in processing conflicting motor impulses, conflicting valence information and conflicts with action–outcome learning biases.

Furthermore, the results are limited by the relative variability in placement of electrodes across STN subregions. That is, in the current study, dorsal and ventral electrodes were selected for each individual patient. This allowed us to make a relative comparison of DBS effects along the gradient of the STN in terms of reward-based learning outcomes, but the relative dorsal or ventral position of the contacts across individuals could impact the outcomes. In a *post hoc* analysis, we tested this by correlating the electrode coordinates of the inferior–superior axes for dorsal and ventral electrodes with the Pavlovian biases. None of those correlations were significant (see Supplementary Table 3). Future studies aiming to understand the underlying functional circuitry of the STN could prospectively use normative connectomes (i.e. LeadDBS)^73,76^ to delineate stimulation effects on motor and valence networks more precisely.

It is also worth noting that the exact mechanism of DBS on local and network-wide levels remains an enigma; for example, stimulation could function similar to lesion by inhibiting neurons close to the electrode, lower overactive beta-band oscillations between STN and cortex, may cause antidromic action potentials to cortex or alter the dynamics of neurotransmitters.^77-79^ Although currently limited to animal studies, optogenetic approaches allow control over specific STN cell populations, which has yielded more detailed insight into the neural mechanism of DBS on motor deficits in Parkinson’s disease.^9,80^ Similar techniques could be used in the context of action–outcome learning paradigms to understand STN-DBS effects on reward-related behaviour.

Finally, we recognize the following limitations in our study design: there was no DBS OFF condition to compare the focused stimulation with, and our findings are based on a small sample size. Accordingly, future studies should strive to incorporate a larger group of participants and include a control group OFF DBS.

## Conclusion

The current study contributes to a growing literature on how neuromodulation in the basal ganglia circuitry impacts stimulus–action–outcome learning, a fundamental aspect of human cognition that influences daily learning and decision-making. Beyond Parkinson’s disease, understanding neuromodulation effects on action–outcome learning and Pavlovian biases could be relevant to a range of neurologic and neuropsychiatric diseases (OCD, addiction, depression and Huntington’s disease that could benefit from DBS^13,81,82^).

## Supplementary Material

fcae111_Supplementary_Data

## Data Availability

Anonymized data will be available upon reasonable request by a qualified investigator.

## References

[1] Guitart-Masip M, Nuys QJM, Fuentemilla L, Dayan P, Duzel E, Dolan RJ. Go and no-go learning in reward and punishment: Interactions between affect and effect. Neuroimage. 2012;62(1):154–166. 22548809 10.1016/j.neuroimage.2012.04.024 PMC3387384

[2] Ridderinkhof KR, Forstmann BU, Wylie SA, Burle B, van den Wildenberg WPM. Neurocognitive mechanisms of action control: Resisting the call of the Sirens. Wiley Interdiscip Rev Cogn Sci. 2011;2(2):174–192. 26302009 10.1002/wcs.99

[3] Hershberger WA . An approach through the looking-glass. Anim Learn Behav. 1986;14(4):443–451.

[4] DeLong MR, Wichmann T. Circuits and circuit disorders of the basal ganglia. Arch Neurol. 2007;64(1):20–24. 17210805 10.1001/archneur.64.1.20

[5] Dayan P, Niv Y, Seymour B, Daw ND. The misbehavior of value and the discipline of the will. Neural Netw. 2006;19(8):1153–1160. 16938432 10.1016/j.neunet.2006.03.002

[6] Guitart-Masip M, Duzel E, Dolan R, Dayan P. Action versus valence in decision making. Trends Cogn Sci. 2014;18(4):194–202. 24581556 10.1016/j.tics.2014.01.003 PMC3989998

[7] Elliott R, Newman JL, Longe OA, Deakin JFW. Instrumental responding for rewards is associated with enhanced neuronal response in subcortical reward systems. Neuroimage. 2004;21(3):984–990. 15006665 10.1016/j.neuroimage.2003.10.010

[8] Tricomi EM, Delgado MR, Fiez JA. Modulation of caudate activity by action contingency. Neuron. 2004;41(2):281–292. 14741108 10.1016/s0896-6273(03)00848-1

[9] Gunaydin LA, Yizhar O, Berndt A, Sohal VS, Deisseroth K, Hegemann P. Ultrafast optogenetic control. Nat Neurosci. 2010;13(3):387–392. 20081849 10.1038/nn.2495

[10] Robbins TW, Cools R. Cognitive deficits in Parkinson’s disease: A cognitive neuroscience perspective. Mov Disord. 2014;29(5):597–607. 24757109 10.1002/mds.25853

[11] Grahn JA, Parkinson JA, Owen AM. The role of the basal ganglia in learning and memory: Neuropsychological studies. Behav Brain Res. 2009;199(1):53–60. 19059285 10.1016/j.bbr.2008.11.020

[12] Albrecht MA, Waltz JA, Cavanagh JF, Frank MJ, Gold JM. Reduction of Pavlovian bias in schizophrenia: Enhanced effects in clozapine-administered patients. PLoS One. 2016;11(4):e0152781. 27044008 10.1371/journal.pone.0152781 PMC4833478

[13] Peng ZW, He LN, Wen RZ, Verguts T, Seger CA, Chen Q. Obsessive-compulsive disorder is characterized by decreased Pavlovian influence on instrumental behavior. PLoS Comput Biol. 2022;18(10):e1009945. 36215326 10.1371/journal.pcbi.1009945 PMC9584381

[14] Frank MJ . Dynamic dopamine modulation in the basal ganglia: A neurocomputational account of cognitive deficits in medicated and nonmedicated Parkinsonism. J Cogn Neurosci. 2005;17(1):51–72. 15701239 10.1162/0898929052880093

[15] Eisinger RS, Scott BM, Le A, et al Pavlovian bias in Parkinson’s disease: An objective marker of impulsivity that modulates with deep brain stimulation. Sci Rep. 2020;10(1):13448. 32778775 10.1038/s41598-020-69760-y PMC7417529

[16] Van Wouwe NC, Claassen DO, Neimat JS, Kanoff KE, Wylie SA. Dopamine selectively modulates the outcome of learning unnatural action-valence associations. J Cogn Neurosci. 2017;29(5):816–826. 28129053 10.1162/jocn_a_01099 PMC5493525

[17] Wagenbreth C, Zaehle T, Galazky I, et al Deep brain stimulation of the subthalamic nucleus modulates reward processing and action selection in Parkinson patients. J Neurol. 2015;262(6):1541–1547. 25929662 10.1007/s00415-015-7749-9

[18] Chopra A, Tye SJ, Lee KH, et al Underlying neurobiology and clinical correlates of mania status after subthalamic nucleus deep brain stimulation in Parkinson’s disease: A review of the literature. J Neuropsych Clin Neurosci. 2012;24(1):102–110. 10.1176/appi.neuropsych.10070109 PMC3570815 22450620

[19] Witjas T, Baunez C, Henry JM, et al Addiction in Parkinson’s disease: Impact of subthalamic nucleus deep brain stimulation. Mov Disord. 2005;20(8):1052–1055. 15858803 10.1002/mds.20501

[20] Aron AR, Herz DM, Brown P, Forstmann BU, Zaghloul K. Frontosubthalamic circuits for control of action and cognition. J Neurosci. 2016;36(45):11489–11495. 27911752 10.1523/JNEUROSCI.2348-16.2016 PMC5125216

[21] Cavanagh JF, Wiecki TV, Cohen MX, et al Subthalamic nucleus stimulation reverses mediofrontal influence over decision threshold. Nat Neurosci. 2011;14(11):1462–1467. 21946325 10.1038/nn.2925 PMC3394226

[22] Haynes WI, Haber SN. The organization of prefrontal-subthalamic inputs in primates provides an anatomical substrate for both functional specificity and integration: Implications for basal ganglia models and deep brain stimulation. J Neurosci. 2013;33(11):4804–4814. 23486951 10.1523/JNEUROSCI.4674-12.2013 PMC3755746

[23] Jahanshahi M, Obeso I, Baunez C, Alegre M, Krack P. Parkinson’s disease, the subthalamic nucleus, inhibition, and impulsivity. Mov Disord. 2015;30(2):128–140. 25297382 10.1002/mds.26049

[24] Zaghloul KA, Weidemann CT, Lega BC, Jaggi JL, Baltuch GH, Kahana MJ. Neuronal activity in the human subthalamic nucleus encodes decision conflict during action selection. J Neurosci. 2012;32(7):2453–2460. 22396419 10.1523/JNEUROSCI.5815-11.2012 PMC3296967

[25] Zavala B, Jang A, Trotta M, Lungu CI, Brown P, Zaghloul KA. Cognitive control involves theta power within trials and beta power across trials in the prefrontal-subthalamic network. Brain. 2018;141(12):3361–3376. 30358821 10.1093/brain/awy266 PMC6272116

[26] Frank MJ, Samanta J, Moustafa AA, Sherman SJ. Hold your horses: Impulsivity, deep brain stimulation, and medication in parkinsonism. Science. 2007;318(5854):1309–1312. 17962524 10.1126/science.1146157

[27] Bogacz R, Gurney K. The basal ganglia and cortex implement optimal decision making between alternative actions. Neural Comput. 2007;19(2):442–477. 17206871 10.1162/neco.2007.19.2.442

[28] Wiecki TV, Sofer I, Frank MJ. HDDM: Hierarchical Bayesian estimation of the drift-diffusion model in Python. Front Neuroinform. 2013;7:14. 23935581 10.3389/fninf.2013.00014 PMC3731670

[29] Baunez C, Robbins TW. Bilateral lesions of the subthalamic nucleus induce multiple deficits in an attentional task in rats. Eur J Neurosci. 1997;9(10):2086–2099. 9421169 10.1111/j.1460-9568.1997.tb01376.x

[30] Jahanshahi M, Ardouin CM, Brown RG, et al The impact of deep brain stimulation on executive function in Parkinson’s disease. Brain. 2000;123(Pt 6):1142–1154. 10825353 10.1093/brain/123.6.1142

[31] Witt K, Pulkowski U, Herzog J, et al Deep brain stimulation of the subthalamic nucleus improves cognitive flexibility but impairs response inhibition in Parkinson disease. Arch Neurol. 2004;61(5):697–700. 15148146 10.1001/archneur.61.5.697

[32] Hershey T, Revilla FJ, Wernle A, Gibson PS, Dowling JL, Perlmutter JS. Stimulation of STN impairs aspects of cognitive control in PD. Neurology. 2004;62(7):1110–1114. 15079009 10.1212/01.wnl.0000118202.19098.10

[33] Wylie SA, Ridderinkhof KR, Elias WJ, et al Subthalamic nucleus stimulation influences expression and suppression of impulsive behaviour in Parkinson’s disease. Brain. 2010;133(Pt 12):3611–3624. 20861152 10.1093/brain/awq239 PMC2995881

[34] Hershey T, Campbell MC, Videen TO, et al Mapping Go-No-Go performance within the subthalamic nucleus region. Brain. 2010;133:3625–3634. 20855421 10.1093/brain/awq256 PMC2995882

[35] Ballanger B, van Eimeren T, Moro E, et al Stimulation of the subthalamic nucleus and impulsivity: Release your horses. Ann Neurol. 2009;66(6):817–824. 20035509 10.1002/ana.21795 PMC2972250

[36] Eagle DM, Baunez C. Is there an inhibitory-response-control system in the rat? Evidence from anatomical and pharmacological studies of behavioral inhibition. Neurosci Biobehav Rev. 2010;34(1):50–72. 19615404 10.1016/j.neubiorev.2009.07.003 PMC2789250

[37] Plantinga BR, Temel Y, Duchin Y, et al Individualized parcellation of the subthalamic nucleus in patients with Parkinson’s disease with 7T MRI. Neuroimage. 2018;168:403–411. 27688203 10.1016/j.neuroimage.2016.09.023 PMC5479742

[38] Mallet L, Schupbach M, N'Diaye K, et al Stimulation of subterritories of the subthalamic nucleus reveals its role in the integration of the emotional and motor aspects of behavior. Proc Natl Acad Sci U S A. 2007;104(25):10661–10666. 17556546 10.1073/pnas.0610849104 PMC1965569

[39] Okun MS, Fernandez HH, Wu SS, et al Cognition and mood in Parkinson’s disease in subthalamic nucleus versus globus pallidus interna deep brain stimulation: The COMPARE trial. Ann Neurol. 2009;65(5):586–595. 19288469 10.1002/ana.21596 PMC2692580

[40] Accolla EA, Pollo C. Mood effects after deep brain stimulation for Parkinson’s disease: An update. Front Neurol. 2019;10:617. 31258509 10.3389/fneur.2019.00617 PMC6587122

[41] Greenhouse I, Gould S, Houser M, Hicks G, Gross J, Aron AR. Stimulation at dorsal and ventral electrode contacts targeted at the subthalamic nucleus has different effects on motor and emotion functions in Parkinson’s disease. Neuropsychologia. 2011;49(3):528–534. 21184765 10.1016/j.neuropsychologia.2010.12.030

[42] Buot A, Welter ML, Karachi C, et al Processing of emotional information in the human subthalamic nucleus. J Neurol Neurosurg Psychiatry. 2013;84(12):1331–1338. 23100448 10.1136/jnnp-2011-302158

[43] van Wouwe NC, Pallavaram S, Phibbs FT, et al Focused stimulation of dorsal subthalamic nucleus improves reactive inhibitory control of action impulses. Neuropsychologia. 2017;99:37–47. 28237741 10.1016/j.neuropsychologia.2017.02.016 PMC5493526

[44] van Wouwe NC, Neimat JS, van den Wildenberg WPM, et al Subthalamic nucleus subregion stimulation modulates inhibitory control. Cereb Cortex Commun. 2020;1(1):tgaa083. 33381760 10.1093/texcom/tgaa083 PMC7750129

[45] White-Dzuro GA, Lake W, Eli IM, Neimat JS. Novel approach to securing deep brain stimulation leads: Technique and analysis of lead migration, breakage, and surgical infection. Stereotact Funct Neurosurg. 2016;94(1):18–23. 26882003 10.1159/000442893

[46] D'Haese PF, Pallavaram S, Li R, et al CranialVault and its CRAVE tools: A clinical computer assistance system for deep brain stimulation (DBS) therapy. Med Image Anal. 2012;16(3):744–753. 20732828 10.1016/j.media.2010.07.009 PMC3021628

[47] Liu Y, 'Haese D, Newton PF, Dawant AT, M B. Generation of human thalamus atlases from 7T data and application to intrathalamic nuclei segmentation in clinical 3T T1-weighted images. Magn Reson Imaging. 2020;65:114–128. 31629074 10.1016/j.mri.2019.09.004

[48] Rohde GK, Aldroubi A, Dawant BM. The adaptive bases algorithm for intensity-based nonrigid image registration. IEEE Trans Med Imaging. 2003;22(11):1470–1479. 14606680 10.1109/TMI.2003.819299

[49] Butson CR, McIntyre CC. Current steering to control the volume of tissue activated during deep brain stimulation. Brain Stimul. 2008;1(1):7–15. 19142235 10.1016/j.brs.2007.08.004 PMC2621081

[50] Justin Rossi P, Peden C, Castellanos O, Foote KD, Gunduz A, Okun MS. The human subthalamic nucleus and globus pallidus internus differentially encode reward during action control. Hum Brain Mapp. 2017;38(4):1952–1964. 28130916 10.1002/hbm.23496 PMC6867118

[51] Temperli P, Ghika J, Villemure JG, Burkhard PR, Bogousslavsky J, Vingerhoets FJG. How do parkinsonian signs return after discontinuation of subthalamic DBS? Neurology. 2003;60(1):78–81. 12525722 10.1212/wnl.60.1.78

[52] Lopiano L, Torre E, Benedetti F, et al Temporal changes in movement time during the switch of the stimulators in Parkinson’s disease patients treated by subthalamic nucleus stimulation. Eur Neurol. 2003;50(2):94–99. 12944714 10.1159/000072506

[53] Stoltzfus JC . Logistic regression: A brief primer. Acad Emerg Med. 2011;18(10):1099–1104. 21996075 10.1111/j.1553-2712.2011.01185.x

[54] R: A Language and Environment for Statistical Computing. R Foundation for Statistical Computing, Vienna. https://www.R-project.org. 2021.

[55] Vachez YM, Creed MC. Deep brain stimulation of the subthalamic nucleus modulates reward-related behavior: A systematic review. Front Hum Neurosci. 2020;14:578564. 33328933 10.3389/fnhum.2020.578564 PMC7714911

[56] Zoon TJ, de Bie RM, Schuurman PR, van den Munckhof P, Denys D, Figee M. Resolution of apathy after dorsal instead of ventral subthalamic deep brain stimulation for Parkinson’s disease. J Neurol. 2019;266(5):1267–1269. 30788615 10.1007/s00415-019-09232-0

[57] Le Jeune F, Drapier D, Bourguignon A, et al Subthalamic nucleus stimulation in Parkinson disease induces apathy: A PET study. Neurology. 2009;73(21):1746–1751. 19933975 10.1212/WNL.0b013e3181c34b34

[58] Hack N, Akbar U, Thompson-Avila A, et al Impulsive and compulsive behaviors in Parkinson Study Group (PSG) centers performing deep brain stimulation surgery. J Parkinsons Dis. 2014;4(4):591–598. 25035311 10.3233/JPD-140357

[59] Zahodne LB, Susatia F, Bowers D, et al Binge eating in Parkinson’s disease: Prevalence, correlates and the contribution of deep brain stimulation. J Neuropsychiatry Clin Neurosci. 2011;23(1):56–62. 21304139 10.1176/appi.neuropsych.23.1.56 PMC3075093

[60] Moum SJ, Price CC, Limotai N, et al Effects of STN and GPi deep brain stimulation on impulse control disorders and dopamine dysregulation syndrome. PLoS One. 2012;7(1):e29768. 22295068 10.1371/journal.pone.0029768 PMC3266249

[61] Cartmill T, Skvarc D, Bittar R, McGillivray J, Berk M, Byrne LK. Deep brain stimulation of the subthalamic nucleus in Parkinson’s disease: A meta-analysis of mood effects. Neuropsychol Rev. 2021;31(3):385–401. 33606174 10.1007/s11065-020-09467-z

[62] Mosley PE, Paliwal S, Robinson K, et al The structural connectivity of subthalamic deep brain stimulation correlates with impulsivity in Parkinson’s disease. Brain. 2020;143(7):2235–2254. 32568370 10.1093/brain/awaa148

[63] Mosley PE, Smith D, Coyne T, Silburn P, Breakspear M, Perry A. The site of stimulation moderates neuropsychiatric symptoms after subthalamic deep brain stimulation for Parkinson’s disease. NeuroImage Clinical. 2018;18:996–1006. 29876284 10.1016/j.nicl.2018.03.009 PMC5988013

[64] Weintraub D, Koester J, Potenza MN, et al Impulse control disorders in Parkinson disease: A cross-sectional study of 3090 patients. Arch Neurol. 2010;67(5):589–595. 20457959 10.1001/archneurol.2010.65

[65] Forstmann BU, Dutilh G, Brown S, et al Striatum and pre-SMA facilitate decision-making under time pressure. Proc Natl Acad Sci U S A. 2008;105(45):17538–17542. 18981414 10.1073/pnas.0805903105 PMC2582260

[66] Forstmann BU, van den Wildenberg WP, Ridderinkhof KR. Neural mechanisms, temporal dynamics, and individual differences in interference control. J Cogn Neurosci. 2008;20(10):1854–1865. 18370596 10.1162/jocn.2008.20122

[67] David FJ, Munoz MJ, Corcos DM. The effect of STN DBS on modulating brain oscillations: Consequences for motor and cognitive behavior. Exp Brain Res. 2020;238(7–8):1659–1676. 32494849 10.1007/s00221-020-05834-7 PMC7415701

[68] Chen W, de Hemptinne C, Miller AM, et al Prefrontal-subthalamic hyperdirect pathway modulates movement inhibition in humans. Neuron. 2020;106(4):579–588.e3. 32155442 10.1016/j.neuron.2020.02.012 PMC7274135

[69] Pasquereau B, Turner RS. A selective role for ventromedial subthalamic nucleus in inhibitory control. Elife. 2017;6:e31627. 29199955 10.7554/eLife.31627 PMC5730370

[70] Miletic S, Keuken MC, Mulder MJ, Trampel R, de Hollander G, Forstmann BU. 7T functional MRI finds no evidence for distinct functional subregions in the subthalamic nucleus during a speeded decision-making task. Cortex. 2022;155:162–188. 35994782 10.1016/j.cortex.2022.06.014

[71] Keuken MC, Uylings HB, Geyer S, Schafer A, Turner R, Forstmann BU. Are there three subdivisions in the primate subthalamic nucleus? Front Neuroanat. 2012;6:14. 22590455 10.3389/fnana.2012.00014 PMC3349268

[72] Howell B, Gunalan K, McIntyre CC. A driving-force predictor for estimating pathway activation in patient-specific models of deep brain stimulation. Neuromodulation. 2019;22(4):403–415. 30775834 10.1111/ner.12929 PMC6579680

[73] Horn A, Li NF, Dembek TA, et al Lead-DBS v2: Towards a comprehensive pipeline for deep brain stimulation imaging. Neuroimage. 2019;184:293–316. 30179717 10.1016/j.neuroimage.2018.08.068 PMC6286150

[74] Petersen MV, Mlakar J, Haber SN, et al Holographic reconstruction of axonal pathways in the human brain. Neuron. 2019;104(6):1056–1064.e1053. 31708306 10.1016/j.neuron.2019.09.030 PMC6948195

[75] Noecker AM, Frankemolle-Gilbert AM, Howell B, et al StimVision v2: Examples and applications in subthalamic deep brain stimulation for Parkinson’s disease. Neuromodulation. 2021;24(2):248–258. 33389779 10.1111/ner.13350 PMC8581744

[76] Horn A, Reich M, Vorwerk J, et al Connectivity predicts deep brain stimulation outcome in Parkinson disease. Ann Neurol. 2017;82(1):67–78. 28586141 10.1002/ana.24974 PMC5880678

[77] Chiken S, Nambu A. Mechanism of deep brain stimulation: Inhibition, excitation, or disruption? Neuroscientist. 2016;22(3):313–322. 25888630 10.1177/1073858415581986 PMC4871171

[78] Herrington TM, Cheng JJ, Eskandar EN. Mechanisms of deep brain stimulation. J Neurophysiol. 2016;115(1):19–38. 26510756 10.1152/jn.00281.2015 PMC4760496

[79] Alosaimi F, Boonstra JT, Tan S, Temel Y, Jahanshahi A. The role of neurotransmitter systems in mediating deep brain stimulation effects in Parkinson’s disease. Front Neurosci. 2022;16:998932. 36278000 10.3389/fnins.2022.998932 PMC9579467

[80] Yu CX, Cassar IR, Sambangi J, Grill WM. Frequency-specific optogenetic deep brain stimulation of subthalamic nucleus improves Parkinsonian motor behaviors. J Neurosci. 2020;40(22):4323–4334. 32312888 10.1523/JNEUROSCI.3071-19.2020 PMC7252487

[81] Chen K, Garbusow M, Sebold M, Zech HG, Zimmermann U, Heinz A. Automatic approach behaviors in alcohol dependence: Does a cognitive bias modification training affect pavlovian-to-instrumental transfer effects? Neuropsychobiology. 2022;81(5):387–402. 36404705 10.1159/000526805

[82] Nord CL, Lawson RP, Huys QJM, Pilling S, Roiser JP. Depression is associated with enhanced aversive Pavlovian control over instrumental behaviour. Sci Rep. 2018;8(1):12582. 30135491 10.1038/s41598-018-30828-5 PMC6105578

