# Supplementary material for: Focused stimulation of dorsal versus ventral subthalamic nucleus enhances action–outcome learning in patients with Parkinson’s disease
Source: Brain Commun. 2024 Apr 2;6(2):fcae111. doi: 10.1093/braincomms/fcae111 (PMC11032193; doi:10.1093/braincomms/fcae111)
Supplement: fcae111_Supplementary_Data [file fcae111_supplementary_data.docx]

SUPPLEMENTARY INFORMATION

**Participants**

Participants were excluded from participation if their clinical history aligned with any of the following: 1) comorbid neurological condition(s) aside from PD such as essential tremor, stroke, epilepsy, etc., 2) schizophrenia or bipolar affective disorder, 3) severe, treatment-resistant mood disorders, or 4) additional medical condition directly impacting cognition. Comorbid depression and/or anxiety did not meet exclusionary criteria if symptoms were appropriately managed, stable, and not beyond that of low moderate severity at the time of entry. In other words, meeting similar requirements for surgical candidacy as defined by consensus conference reviews, neuropsychological interviews, and questionnaires. ^1^ It was the practice of the multidisciplinary surgical team to exclude patients with significant or early-stage dementia based on a comprehensive neuropsychological report from surgery. These patients were therefore not considered for this study. If mild cognitive difficulties were present, participants had to score a minimum of 25 on the Mini Mental State Examination (MMSE)^2^ to be eligible for inclusion. All participants had corrected-to-normal vision at the time of the study.

The participants had undergone awake neurosurgical DBS using microelectrode recordings as part of the normal clinical care using standard stereotactic techniques. To ensure optimal lead placement, microelectrode recordings, and motor testing were performed intraoperatively. ^3^ The Activa PC Medtronic neurostimulator (Medtronic Inc.) was used.

Supplementary Table 1. Clinical stimulation settings (mean and standard deviation)

|  | **Clinical DBS settings** |  |
| --- | --- | --- |
| **Sample Size (N)** | 12 | |
| **Left** |  | |
| **Voltage (V)** | 2.58 (0.26) | |
| **Frequency (Hz)** | 125.83 (2.60) | |
| **Pulse width (ms)** | 59.08 (4.41) | |
| **Right** |  | |
| **Voltage (V)** | 2.30 (0.28) | |
| **Frequency (Hz)** | 125.83 (2.60) | |
| **Pulse width (ms)** | 61.58 (5.11) |  |

Supplementary Table 2. Contacts used with clinical stimulation settings.

| **Subject ID** | **Left** | **Right** |
| --- | --- | --- |
|  |  |  |
| **1** | C+3- | C+2- |
| **2** | C+2- | C+2- |
| **3** | C+2- | C+3- |
| **4** | C+3- | C+3- |
| **5** | C+2- | C+2- |
| **6** | C+3- | C+1- |
| **7** | C+2-3- | C+1- |
| **8** | C+1- | C+2- |
| **9** | 3+2-1- | 3+2-1- |
| **10** | C+1- | C+2- |
| **11** | C+2- | C+2- |
| **12** | C+2- | C+2- |

Supplementary Table 3. Pavlovian reward and punishment bias correlated with the electrode coordinate inferior-superior axis used for focused dorsal and ventral stimulation.

| **Pavlovian bias** | **L Dorsal** | | **R Dorsal** | **L Ventral** | **R Ventral** |
| --- | --- | --- | --- | --- | --- |
| **Dorsal reward** | r=.12,p=.72 | | r=.20 p=.55 |  |  |
| **Dorsal punishment** | r=.005 p=.99 | | r=.30 p=.36 |  |  |
| **Ventral reward** |  | |  | r=.43 p=.18 | r=.15 p=.65 |
| **Ventral punishment** |  |  | | r=-.32 p=.34 | r=.18 p=.60 |

Supplementary References

1. Radloff LS. The CES-D Scale: A self-report depression scale for research in the general population. *Applied Psychological Measurement.* 1977;1(3):385-401.

2. Folstein MF, Folstein SE, McHugh PR. "Mini-mental state". A practical method for grading the cognitive state of patients for the clinician. *J Psychiatr Res.* 1975;12(3):189-198.

3. Konrad PE, Neimat JS, Yu H, et al. Customized, miniature rapid-prototype stereotactic frames for use in deep brain stimulator surgery: initial clinical methodology and experience from 263 patients from 2002 to 2008. *Stereotact Funct Neurosurg.* 2011;89(1):34-41.
